# Use of Multiplex Allele-Specific Polymerase Chain Reaction (MAS-PCR) to Detect Multidrug-Resistant Tuberculosis in Panama

**DOI:** 10.1371/journal.pone.0040456

**Published:** 2012-07-06

**Authors:** Bing-Shao Chia, Fedora Lanzas, Dalin Rifat, Aubrey Herrera, Elizabeth Y. Kim, Christine Sailer, Edith Torres-Chavolla, Purvaja Narayanaswamy, Viktor Einarsson, Jaime Bravo, Juan M. Pascale, Thomas R. Ioerger, James C. Sacchettini, Petros C. Karakousis

**Affiliations:** 1 Department of Medicine, Johns Hopkins University School of Medicine, Baltimore, Maryland, United States of America; 2 Department of Genomics and Proteomics, Instituto Conmemorativo Gorgas de Estudios de la Salud, ICGES, Panamá, República de Panamá; 3 Department of International Health, Johns Hopkins Bloomberg School of Public Health, Baltimore, Maryland, United States of America; 4 Department of Computer Science, Texas A&M University, College Station, Texas, United States of America; 5 Department of Biochemistry/Biophysics, Texas A&M University, College Station, Texas, United States of America; Fundació Institut d'Investigació en Ciències de la Salut Germans Trias i Pujol. Universitat Autònoma de Barcelona. CIBERES, Spain

## Abstract

The frequency of individual genetic mutations conferring drug resistance (DR) to *Mycobacterium tuberculosis* has not been studied previously in Central America, the place of origin of many immigrants to the United States. The current gold standard for detecting multidrug-resistant tuberculosis (MDR-TB) is phenotypic drug susceptibility testing (DST), which is resource-intensive and slow, leading to increased MDR-TB transmission in the community. We evaluated multiplex allele-specific polymerase chain reaction (MAS-PCR) as a rapid molecular tool to detect MDR-TB in Panama. Based on DST, 67 MDR-TB and 31 drug-sensitive clinical isolates were identified and cultured from an archived collection. Primers were designed to target five mutation hotspots that confer resistance to the first-line drugs isoniazid and rifampin, and MAS-PCR was performed. Whole-genome sequencing confirmed DR mutations identified by MAS-PCR, and provided frequencies of genetic mutations. DNA sequencing revealed 70.1% of MDR strains to have point mutations at codon 315 of the *katG* gene, 19.4% within *mabA-inhA* promoter, and 98.5% at three hotspots within *rpoB*. MAS-PCR detected each of these mutations, yielding 82.8% sensitivity and 100% specificity for isoniazid resistance, and 98.4% sensitivity and 100% specificity for rifampin resistance relative to DST. The frequency of individual DR mutations among MDR strains in Panama parallels that of other TB-endemic countries. The performance of MAS-PCR suggests that it may be a relatively inexpensive and technically feasible method for rapid detection of MDR-TB in developing countries.

## Introduction

Tuberculosis (TB) remains a global scourge, causing 8.8 million new infections and 1.45 million deaths in 2010 [1]. The emergence of multidrug-resistant TB (MDR-TB), defined as resistance of *Mycobacterium tuberculosis* to the first-line drugs isoniazid (INH) and rifampin (RIF), poses a serious threat to global TB control. MDR-TB treatment requires second-line drugs, which are less effective, more expensive, and more toxic [2]. Accordingly, MDR-TB is associated with higher treatment failure rates, as well as increased transmission and mortality. The World Health Organization estimated that approximately 650,000 of the 12 million prevalent TB cases in 2010 represented MDR-TB [1]. In Central America, the annual number of confirmed MDR-TB cases, likely a gross underestimate, ranged from 0–50 per country in 2008–2010, with the exception of Guatemala, which reported 230 MDR-TB cases in 2009 [1]. In Panama, of the approximately 1800 prevalent TB cases in 2010, 75 underwent drug susceptibility testing and 10 were confirmed to have MDR-TB [1].

Although the frequency of individual genetic mutations conferring drug resistance (DR) has been reported for some South American countries [3], [4], [5], their frequency has not been studied in Central America. Central Americans represent the fastest growing sector of the Latin American population in the U.S. over the last decade [6], and pose an elevated risk of TB relative to the U.S.-born population [7]. As particular mutations are associated with varying degrees of resistance [8], the mutation profile of MDR-TB strains originating from this region could help guide therapeutic decisions and aid in developing rapid molecular assays for diagnosing MDR-TB.

INH resistance mutations are primarily found in *katG*, encoding the catalase-peroxidase enzyme responsible for activating INH [9], *inhA*, which encodes the molecular target InhA of the activated drug, and the promoter region of the *mabA-inhA* operon, resulting in InhA overexpression [10]. Mutations in *katG* and *inhA* account for 50–95% and 15–34%, respectively, of INH-resistant clinical isolates [10]. The substitution S315T comprises the majority of *katG*-associated mutations and confers high-level INH resistance (MIC>5 µg/ml), while the −15C→T *mabA-inhA* promoter mutation accounts for the majority of *inhA*-associated mutations [10] and confers low-level resistance (MIC<1 µg/ml).

RIF resistance is primarily caused by mutations in *rpoB*, which encodes the β-subunit of RNA polymerase targeted by RIF [10]. Approximately 95% of RIF-resistant clinical isolates contain point mutations clustered in an 81 bp RIF resistance-determining region (RRDR) between codons 507–533, with the three most common mutations located at codons 516, 526 and 531 [10]. The frequency at each codon exhibits substantial geographic variation. For example, the substitutions H526Y and S531L account for 32% and 29% of RIF-resistant clinical isolates in the U.S., while the same mutations represent 12% and 47% of predominantly foreign RIF-resistant isolates [10].

As MDR-TB is difficult to treat, rapid diagnosis and prevention of MDR-TB transmission within the community is imperative. The gold standard to diagnose MDR-TB is culture-based drug susceptibility testing (DST), which significantly delays diagnosis given the slow replication time of *M. tuberculosis*. Due to cost and the requirements for appropriate laboratory infrastructure and technical expertise, DST is not routinely performed in resource-limited settings, but only when DR is suspected based on previous treatment history, lack of clinical response to the standard first-line regimen, or documented exposure to MDR-TB patients. Recently, better understanding of the genetic mechanisms underlying *M. tuberculosis* DR and technological advances have stimulated the development of rapid molecular diagnostics. Multiplex allele-specific PCR (MAS-PCR) permits simultaneous detection of the most common INH and RIF resistance-associated genetic mutations at relatively reduced cost and need for technical expertise [11].

In this study, we report the frequency of the most common INH and RIF mutations among 67 archived MDR-TB clinical isolates from Panama. We also evaluated the ability of the relatively inexpensive and technically feasible method of MAS-PCR to detect these mutations.

## Results

### Drug susceptibility

**Figure 1:**
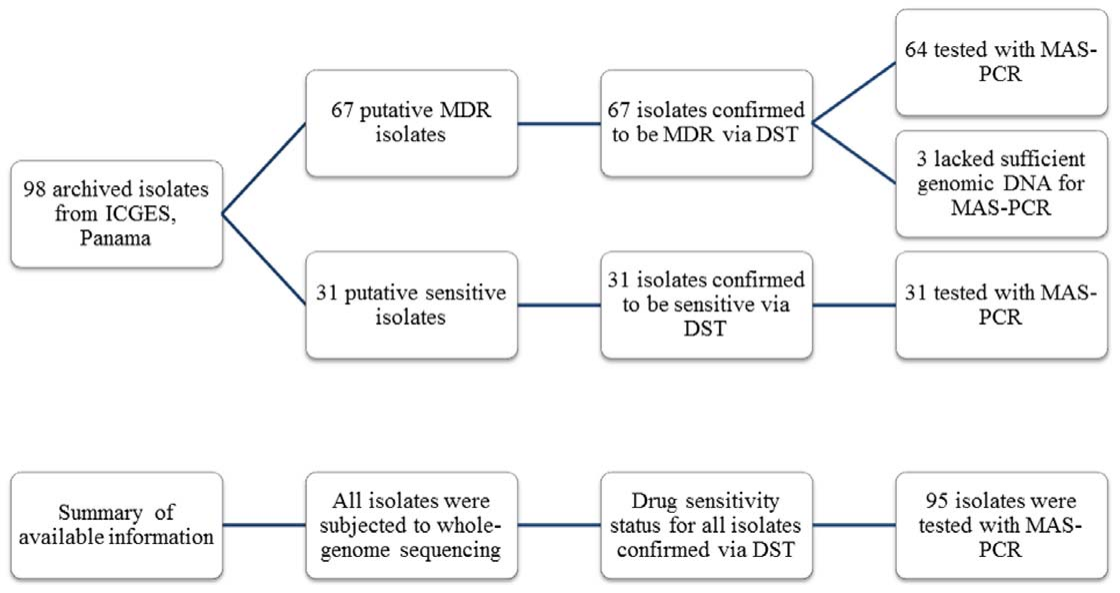
Flow-chart showing available drug susceptibility testing (DST), sequencing, and multiplex allele-specific PCR (MAS-PCR) results for the 98 archived isolates from Panama.

Of the 98 isolates, 31 were susceptible to INH and RIF, and 67 were MDR (Figure 1). No isolate was monodrug-resistant. Multiple DR was detected as follows: 25/67 were resistant to only INH and RIF, 24/67 had additional resistance to streptomycin, and 18/67 were resistant to INH, RIF, streptomycin and ethambutol.

### DNA Sequencing

**Figure 2:**
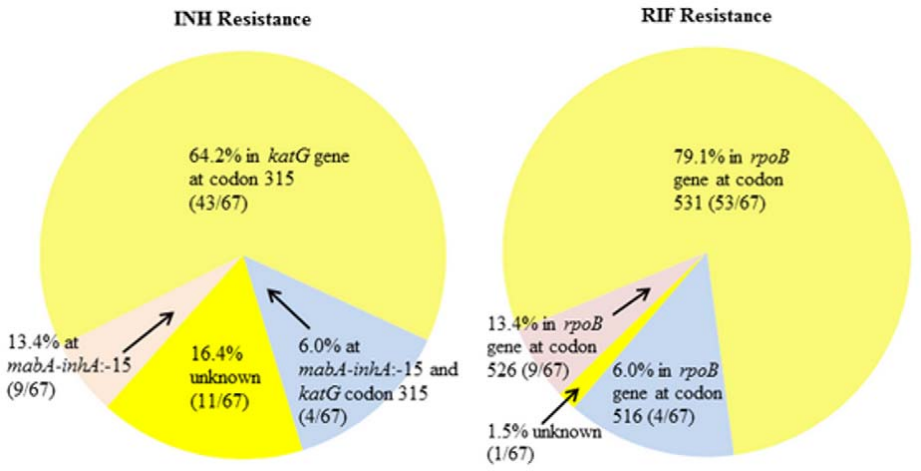
Frequency of the five most common INH and RIF resistance mutations among 67 Panamanian MDR-TB isolates.

DNA sequencing detected point mutations in *katG* codon 315 in 47/67 (70.1%) isolates: 1 isolate contained an S315G (AGC→GGC) mutation, while 46 isolates had the more common S315T mutations (45 AGC→ACC and 1 AGC→ACA). A −15C→T mutation in the *mabA-inhA* operon was identified in 13/67 (19.4%) strains, and four of these isolates (6.0%) had mutations at both locations. RIF resistance-associated mutations were located in the RRDR: 53/67 (79.1%) had S531L mutations, 9/67 (13.4%) had H526D/Y mutations, and 4/67 (6.0%) had D516F/V mutations. No isolate was mutated at more than one of these three loci. Together, mutations at these three loci accounted for 66/67 (98.5%) of RIF-resistant isolates (Figure 2). When compared to the current gold standard DST in detecting dug resistance, DNA sequencing of these five loci yielded 83.6% sensitivity and 100% specificity for INH resistance, and 98.5% sensitivity and 100% specificity for RIF resistance (Table 1).

**Table 1:** Pairwise comparisons of the three drug resistance detection techniques (DST, DNA sequencing, and MAS-PCR) in detecting INH and RIF resistance.

DNA Sequencing vs. DST (total 98 strains)				
DNA Sequencing results	DST results		Sensitivity	Specificity
	No. resistant	No. susceptible		
INH resistance				
Detected	56	0	83.6%	100%
Not detected	11	31		
RIF resistance				
Detected	66	0	98.5%	100%
Not detected	1	31		

MAS-PCR vs. DNA Sequencing (total 95 strains)				
MAS-PCR results	DNA sequencing results		Sensitivity	Specificity
	No. mutated	No. wild-type		
INH resistance				
Detected	53	0	100%	100%
Not detected	0	42		
RIF resistance				
Detected	63	0	100%	100%
Not detected	0	32		

MAS-PCR vs. DST (total 95 strains)				
MAS-PCR results	DST results		Sensitivity	Specificity
	No. resistant	No. susceptible		
INH resistance				
Detected	53	0	82.8%	100%
Not detected	11	31		
RIF resistance				
Detected	63	0	98.4%	100%
Not detected	1	31		

As 11/67 INH-resistant isolates lacked mutations at the two loci mentioned, their DNA sequences were examined for other potential resistance-conferring mutations. One isolate had a large deletion that extends into *katG*, removing the last 320 bases (i.e., 14.4% of the C-terminal end of the predicted protein); two isolates had non-sense mutations at codons 198 and 722, which are predicted to truncate the 740-amino acid KatG; two other isolates contained both AhpC P44R and KatG D94G mutations; and finally, one isolate had a large duplication of approximately 500 kb (Rv3177-Rv3573c).

### Performance of MAS-PCR

**Figure 3:**
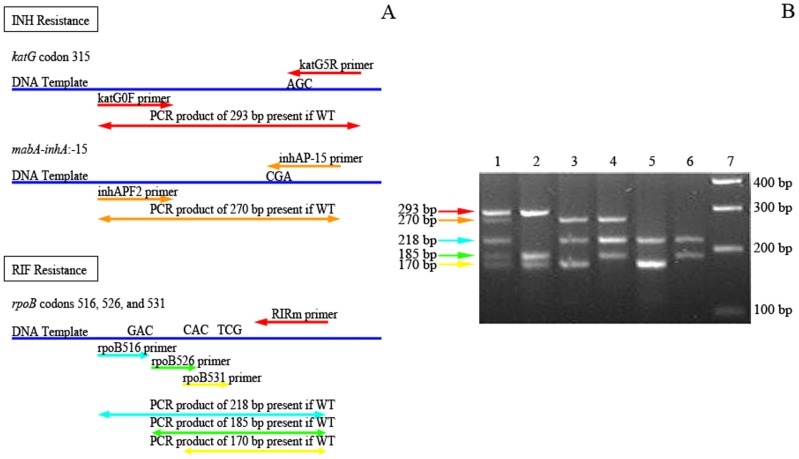
Distinct band patterns indicate drug resistance profile of isolates. A. Loci where each allele-specific primer binds are indicated, along with the expected product size if the locus is wild-type. The two common mutations that confer resistance to INH and the three common mutations that confer resistance to RIF are boxed separately. B. Band patterns indicate drug resistance profile of isolates. Expected PCR products have been color-coded in the same way as in A. Lane 1: H37Rv reference strain (wild-type at all 5 loci); Lane 2: *mabA-inhA* −15C→T and RpoB D516F double mutant; Lane 3: KatG S315T and RpoB H526Y double mutant; Lane 4: KatG S315G and RpoB 531L double mutant; Lane 5: *mabA-inhA* −15C→T, KatG S315T and RpoB H526D triple mutant; lane 6: *mabA-inhA* −15C→T, KatG S315T and RpoB S531L triple mutant; Lane 7: Molecular ladder.

Evaluation of MAS-PCR performance against DNA sequencing and DST was based on 95 isolates, since insufficient DNA was available after genomic sequencing for 3 MDR strains. Distinct banding patterns were obtained for different mutation profiles at the five targeted loci (Figure 3). When compared to sequencing, MAS-PCR had 100% sensitivity and specificity in detecting the common mutations (Table 1). When compared to DST, MAS-PCR had 82.8% sensitivity and 100% specificity for detecting common INH resistance mutations, and 98.4% sensitivity and 100% specificity for detecting common RIF resistance mutations (Table 1). The MAS-PCR results were highly reproducible with the exception of one isolate, for which the MAS-PCR identified an RpoB H526D mutation in some technical replicates but failed to detect this mutation in other replicates, even though the same mutation was consistently detected by MAS-PCR when present in other isolates.

**Table 2:** Primers for MAS-PCR to detect INH and RIF resistance.

Detection targets	Allele-specific primers (5′ – 3′)		Paired primers		PCR product
*katG* gene (at S315)	katG5R	ATACGACCTCGATGCCGCT	katG0F*	GCAGATGGGGCTGATCTACG	293 bp
*mabA-inhA*:-15	inhAP-15*	CACCCCGACAACCTATCG	inhAPF2*	GCGCGGTCAGTTCCACA	270 bp
*rpoB* gene (at D516)	rpoB516	CAGCTGAGCCAATTCATGGAC	RIRm*	TTGACCCGCGCGTACAC	218 bp
*rpoB* gene (at H526)	rpoB526*	CTGTCGGGGTTGACCCA	RIRm*	Same as above.	185 bp
*rpoB* gene (at S531)	rpoB531*	CACAAGCGCCGACTGTC	RIRm*	Same as above.	170 bp

* Primers adopted from [11].

## Discussion

The current study is the first to report the frequency of individual genetic mutations associated with DR among MDR-TB isolates in Central America. It also evaluated the performance characteristics of MAS-PCR as a rapid molecular assay that may decrease delays in diagnosis and treatment of MDR-TB.

We found that 70.1% of the INH-resistant isolates had a KatG S315T/G mutation, and 19.4% had a −15C→T mutation in *mabA-inhA* operon, together accounting for 83.6% of INH-resistant strains in our MDR samples. Our data are concordant with those of similar studies in diverse geographic regions, which documented a frequency of 60.4–93.6% for *katG* codon 315 mutations and 1.8–50% for mutations in the −15 position of the *mabA-inhA* operon [5], [12], [13], [14], [15], [16], [17], [18], [19], [20]. Among Panamanian MDR-TB isolates, *rpoB* codon 531 was the most frequently mutated site (79.1%), consistent with other studies reporting a mutation frequency ranging from 40.1–82.4% at this site in MDR-TB isolates from Mexico, Brazil, Cape Town, China, Russia, Korea, Georgia, Vietnam, India, East Asian countries, and South Africa [5], [12], [13], [14], [15], [16], [17], [18], [19], [20]; *rpoB* mutations at codons 516 (6.0%) and 526 (13.4%) accounted for most of the remaining RIF-resistant strains in our study, similar to what was reported for these sites (2.9–16.7% and 5.9–40%, respectively). The agreement of our results with those derived from highly disparate geographical areas may reflect the international origin of the population of Panama, which lies on a major maritime trade route and whose economy depends heavily on international trade.

MAS-PCR showed excellent performance characteristics, including 100% concordance with DNA sequencing results, as well as 82.8% and 98.4% sensitivity in detecting INH and RIF resistance, respectively. Furthermore, since MAS-PCR experiments were performed in triplicate in two separate labs blinded to the sequencing results, we are confident of the reproducibility of our assay.

MAS-PCR using the original set of *katG* primers [11] failed to detect INH resistance in one isolate containing a S315G mutation (AGC→GGC), leading us to redesign the *katG* allele-specific primer by extending one base at the 3′ end in order to detect this mutation. Importantly, our modification did not decrease the detection rate for the more common AGC→ACC/ACA mutations. Extending this reasoning, we modified the allele-specific primer at *rpoB* codon 516 so that RpoB D516E mutations (previously reported in [21]) could potentially be identified, although none of our isolates harbored this mutation. Notably, our modification did not reduce detection of the more common RpoB D516F/V mutations.

Since RIF monoresistance is rare and >90% of RIF-resistant *M. tuberculosis* are also INH-resistant [22], RIF resistance is commonly used as a surrogate for MDR-TB, including in the recently developed Xpert MTB/RIF assay [23]. Interestingly, MAS-PCR detected an RpoB H526D mutation in some, but not all, technical replicates of one particular isolate, although the identical mutation was always accurately and consistently detected in five other isolates. Since MAS-PCR did accurately identify this mutation in some replicates of the isolate in question, it was counted as a hit instead of as a miss. However, if we were to assume that MAS-PCR failed to detect this mutation, our assay still would have detected 98.4% (62/63) of isolates containing mutations in *rpoB*, and would have identified correctly 96.9% (63/64) of RIF-resistant isolates. It is possible that this isolate comprised a heterogeneous population of organisms with both wild-type and mutated alleles in the *rpoB* gene at codon 526, leading to amplification of the corresponding wild-type PCR product. Whole-genome sequencing data also revealed heterogeneity for this isolate, since among the 91 reads covering this site, only 86% purity was achieved for the nucleotide, much lower than the 99–100% purity achieved for most of the other nucleotides.

DNA sequencing did not reveal any of the common mutations in 16.4% and 1.5% of INH-resistant and RIF-resistant isolates. However, several isolates contained alternative genetic mutations that might account for INH resistance. Three isolates contained mutations that were predicted to yield truncated KatG proteins: one isolate contained a 320 bp deletion at the 3′ end of *katG* (total length of gene: 2223 bp); two isolates had a non-sense mutation at *katG* codons 198 and 722 respectively. The KatG protein C-terminus has been implicated in enzymatic function [9], [24], and a truncation of the C-terminal 41 amino acids inactivated KatG [24]. Although INH resistance has been attributed to KatG truncation [25], the minimum protein length required for INH activation remains uncharacterized. We postulate that truncation of the C-terminal 19 amino acids is sufficient to interfere with subunit-subunit interactions and thus confer INH resistance.

Additionally, two other isolates contained a D94G mutation within the KatG protein. This mutation has not been associated with INH resistance except in one report by Gagneux *et al.* [26], in which the isolate also contained a 34G→A mutation in the *ahpC* promoter. Both isolates here contained both KatG D94G and AhpC P44R mutations. Although AhpC does not appear to directly confer INH resistance, as *ahpC* overexpression in a wild-type reference strain of *M. tuberculosis* does not appreciably increase the MIC of INH, mutations in the *ahpC* promoter region may serve as a useful marker for detecting INH resistance [10]. Whether the KatG D94G mutation leads to inactivation of KatG, and the AhpC P44R mutation compensates for loss of catalase-peroxidase activity requires further study.

Finally, one isolate has a large genomic duplication of approximately 500 kb spanning Rv3177-Rv3573c. The duplication contains the *nat* gene (Rv3566c), whose product has been implicated in inactivating INH, and whose increased expression confers INH resistance [27], [28]. The duplication may lead to an increased expression of *nat* in this isolate, increasing N-acetyltransferase activity and INH inactivation. However, experimental verification of this hypothesis is required.

Five MDR isolates had no obvious mutation that could account for INH resistance. One of these also contained no mutation in *katG*, *inhA* and *rpoB* (except for a synonymous substitution in *rpoB*), suggesting the possibility of alternative DR mechanisms.

One of the limitations of our study was its retrospective nature, which does not allow us to calculate the true prevalence of each resistance mutation among MDR-TB patients in the general population. However, since the Mycobacteriology Lab at the Gorgas Institute serves as the only referral lab for DST in Panama, and since all archived MDR samples were evaluated in this study, we do not believe there was a selection bias. Because archived samples were used, MAS-PCR could only be evaluated from cultured isolates and could not be evaluated directly on patient sputum samples. Future studies will evaluate the limit of detection of MDR-TB among clinical isolates and assess the performance of MAS-PCR directly on sputum samples relative to DST in a prospective manner.

In conclusion, our study shows that the frequency of individual genetic mutations associated with INH and RIF resistance among Panamanian MDR-TB isolates tested closely approximates that of MDR-TB isolates in other geographic locations. The high sensitivity and specificity of MAS-PCR in detecting MDR-TB, along with its low cost relative to other rapid molecular assays and ease of use make it an attractive alternative for rapid detection of MDR relative to DST. Further analysis of the available whole-genome sequences of these archived MDR-TB isolates could reveal novel mutations associated with INH and RIF resistance, as well as mutations that confer resistance to pyrazinamide and second-line drugs. These findings could then be used to further refine the MAS-PCR assay and improve detection of DR in *M. tuberculosis* clinical isolates.

## Materials and Methods

### Ethics statement

This study was approved by the Institutional Review Board of the Johns Hopkins University School of Medicine and the Comité Nacional de Bioética of Panama. The study qualified for an exemption under the DHHS regulations and patient informed consent was not obtained since archived cultures were used, which had been de-identified from any clinical source data.

### M. tuberculosis isolates

Ninety-eight archived clinical isolates collected between 2002 and 2011 at the Department of Mycobacteriology at ICGES, Panama, were studied. DST revealed 67 isolates were MDR-TB and 31 isolates were susceptible to INH and RIF. MAS-PCR was performed on 95 isolates, as 3 isolates had insufficient genomic DNA sample remaining after sequencing (Figure 1).

### Characterization and drug susceptibility test

Sputum samples were decontaminated using a modified version of Petroff's methodology according to the specifications of the Pan-American Health Organization [29]. Decontaminated samples were cultured on Lowenstein-Jensen agar, and an AccuProbe hybridization assay (Gen-Probe) was used to confirm that colonies represented *M. tuberculosis* complex. The isolates were further classified as *M. tuberculosis* using biochemical assays, including nitrate reduction, niacin production, and catalase inhibition at 68**°**C [29]. DST was performed on all isolates using Canetti's multiple proportions method [29], [30] with Lowenstein Jensen media and the following antibiotic concentrations: 0.2 µg/ml INH, 40 µg/ml RIF, 4 µg/ml streptomycin, and 2 µg/ml ethambutol.

### Genomic DNA extraction

Genomic DNA from cultured isolates was extracted using the QIAamp DNA Mini Kit (Qiagen). DNA yield was quantified by absorbance (*A*_260_) using a spectrophotometer (Thermo Fisher Scientific).

### DNA Sequencing

The double-stranded DNA samples were sonicated (Covaris, Inc) to generate fragments, which were converted into blunt ends using T4 DNA polymerase and Klenow enzyme. Next, Klenow Exo was used to extend blunt ends to facilitate ligation to the adaptors (Illumina TruSeq kit, or NuGen). Ligated DNA was size-selected on a 2% agarose gel: fragments of 250–350 bp were excised and recovered with QIAquick Gel Extraction Kit (Qiagen), and PCR-amplified to produce the final DNA library. Samples were multiplexed at 6–12 per lane, and 5 pmol of DNA was loaded onto an additional lane of the sequencing chip for cluster generation. ϕX174 DNA served as a control. The sequencer was operated in paired-end mode, with the reaction running for enough cycles to generate two reads (51–54 bp each) and a multiplexing barcode for each fragment. Each tile on the chip was imaged at different wavelengths to excite the base-specific fluorophores. Image analysis and base-calling was done with the Illumina Offline Basecaller software v1.8, which generated 5–10 million reads per sample.

Comparative genome assembly similar to that described in [31] was performed, using *M. tuberculosis* H37Rv genome as the reference sequence. The reads generated for each strain were mapped against the reference sequence (allowing up to 2 mismatches and no gaps). Paired-end constraints were applied, requiring both reads of a fragment to map to within 500 bp of each other. We identified sites where the consensus base from overlapping reads differed from the expected base in the reference sequence, and sites where coverage was low or had heterogeneous bases, before using a contig-building algorithm to construct local sequences of approximately 200 bp spanning each site to resolve the sites into single nucleotide polymorphisms or insertion-deletions.

### Multiplex allele-specific PCR (MAS-PCR)

A MAS-PCR assay [11] was adapted to detect the five most common INH and RIF-associated mutations, which are located at *katG* codon 315, *mabA-inhA*:-15, and *rpoB* codons 516, 526 and 531 [32], [33], [34]. Five allele-specific primers and three paired primers (Table 2) were combined into a single PCR reaction to yield distinct PCR banding patterns that can accurately detect mutations confirmed by DNA sequencing. In order to improve sensitivity, two allele-specific primers were extended by a base each, placing the most common point mutation at the second base from the 3′ end. If the targeted location is wild-type, the allele-specific fragment is amplified, yielding a visible band. Conversely, a mutation at the targeted location prevents allele-specific amplification. An INH- and RIF-susceptible isolate will yield five bands (293 bp, 270 bp, 218 bp, 180 bp, and 175 bp), while mutations at any of the five locations will result in missing bands (Figure 3).

The reaction mix contained the following primers: rpoB516 (1 pmol), rpoB526 (5 pmol), rpoB531 (32.5 pmol), RIRm (30 pmol), katgOF (1 pmol), katg5R (1 pmol), inhAP-15 (6 pmol) and inhAPF (6 pmol). The other reagents were 10× PCR reaction buffer (2.5 µl), 50 mM MgCl_2_ (2 µl), 10 mM dNTP mixture (0.5 µl), 5 U/µl Taq Polymerase (0.1 µl), DNA template (20 ng), and PCR-grade water to obtain a final volume of 25 µl. The thermocycling parameters consisted of an initial denaturation at 96**°**C for 3 min, 25 cycles of 95**°**C for 50 s, 68**°**C for 40 s, and 72**°**C for 60 s, and a final extension at 72**°**C for 7 min. The PCR products were examined for banding patterns by 2.5% UltraPure Agarose (Invitrogen) gel electrophoresis in 1× Tris-Borate-EDTA buffer under UV light.

### Evaluation of the MAS-PCR assay

DST, DNA sequencing, MAS-PCR results were compared to determine how each technique performed in detecting INH and RIF resistance. Sensitivity and specificity were calculated for each pairwise comparison. The reproducibility of MAS-PCR was also evaluated by performing the assay at each institution in triplicate for each isolate.
